# Normal Myocardial Flow Reserve in HIV-Infected Patients on Stable Antiretroviral Therapy: A Cross-Sectional Study Using Rubidium-82 PET/CT

**DOI:** 10.1097/MD.0000000000001886

**Published:** 2015-10-30

**Authors:** Andreas Knudsen, Thomas E. Christensen, Adam Ali Ghotbi, Philip Hasbak, Anne-Mette Lebech, Andreas Kjær, Rasmus Sejersten Ripa

**Affiliations:** From the Department of Infectious Diseases (AK, A-ML), Copenhagen University Hospital, Hvidovre; and Department of Clinical Physiology, Nuclear Medicine & PET, and Cluster for Molecular Imaging, Rigshospitalet and University of Copenhagen, Copenhagen, Denmark. (AK, TEC, AAG, PH, AK, RSP).

## Abstract

Studies have found HIV-infected patients to be at increased risk of myocardial infarction, which may be caused by coronary microvascular dysfunction. For the first time among HIV-infected patients, we assessed the myocardial flow reserve (MFR) by Rubidium-82 (^82^Rb) positron emission tomography (PET), which can quantify the coronary microvascular function. MFR has proved highly predictive of future coronary artery disease and cardiovascular events in the general population.

In a prospective cross-sectional study, HIV-infected patients all receiving antiretroviral therapy (ART) with full viral suppression and HIV-uninfected controls were scanned using ^82^Rb PET/computed tomography at rest and adenosine-induced stress, thereby obtaining the MFR (stress flow/rest flow), stratified into low ≤1.5, borderline >1.5 to 2.0, or normal >2.0.

Fifty-six HIV-infected patients and 25 controls were included. The HIV-infected patients had a mean age of 53 years (range 37–68 years) with 23% active smokers. The controls had a mean age of 52 years (range 36–68 years) and 26% active smokers. In the HIV-infected group 73% had a normal MFR, 17% borderline, and 10% low values of MFR. Among controls these values were 71%, 19%, and 10%, respectively (*P* = 0.99). However, the HIV-infected group had lower values of stress myocardial blood flow (MBF) (2.63 ± 0.09 mL/g/min vs 2.99 ± 0.14 mL/g/min; *P* = 0.03). We found no evidence of decreased MFR as assessed by ^82^Rb PET among HIV-infected patients on stable ART with full viral suppression compared with HIV-uninfected controls. We did notice a decreased MBF during stress.

## INTRODUCTION

HIV-infected patients appear to be at increased risk of myocardial infarction (MI)^1^ and subclinical coronary atherosclerosis even with low HIV RNA levels and high CD4cell counts.^2,3^ Clinical studies of the brachial and carotid arteries find a higher prevalence of endothelial dysfunction among HIV-infected patients and some studies link these findings with the antiretroviral treatment (ART), specifically in the form of protease inhibitors (PI) and abacavir (ABC).^4–6^ Furthermore, the subtle and complex immune-biologic changes involved in chronic infection with HIV may affect the vascular system.^7,8^ Coronary microvascular dysfunction is thought to reflect the initiation and early changes in the progression toward coronary artery disease (CAD).^9–11^ The use of dynamic positron emission tomography (PET)/computerized tomography (CT) imaging enables the quantification of the absolute myocardial perfusion in mL/g/min by intravenous injection of a perfusion positron-emitting tracer. This leads to the detection of very subtle signs of disease before structural changes occur, thereby guiding a possible preventive therapy. Therefore, for the first time among HIV-infected patients, we assessed the myocardial flow reserve (MFR) by Rubidium-82 (^82^Rb) PET, which is the maximal myocardial blood flow (MBF) during adenosine stress divided by MBF at rest. This ratio depicts the vasodilator function of the coronary circulation, and in the general population MFR assessed by ^82^Rb PET has proved highly predictive of future cardiovascular events.^12–14^

## METHODS

### Participants

Both HIV-infected patients and HIV-uninfected controls were recruited from a previously described cohort.^15^

Inclusion criteria were age >18 years, and for the HIV-infected patients, ART >12 months. Exclusion criteria were asthma, pregnancy, or alcohol or drug abuse hampering the ability to adhere to the protocol.

Fifty-six HIV-infected patients and 25 controls underwent ^82^Rb PET between August 2012 and June 2013.

### Ethics

All patients received oral and written information and gave written consent before inclusion. The study was approved by the Scientific Ethics Committee of the Capital Region of Denmark [protocol number H-C-2008-060] and complied with the declaration of Helsinki.

### PET Imaging

All patients were asked to abstain from caffeine and theophylline-containing substances and medications for 12 hours before imaging. PET myocardial perfusion imaging (MPI) was performed during rest and stress conditions in a single session. For each acquisition, patients received 1110 MBq (±10%) ^82^Rb supplied from a CardioGen-82 Sr-82/Rb-82 generator manufactured for Bracco (Bracco Diagnostics Inc., Princeton, NJ). Both rest and stress images were acquired ECG-gated in list mode for 7 minutes from the start of the ^82^Rb infusion on a Siemens Biograph mCT/PET 128-slice scanner (Siemens Helthcare, Knoxville, TN, USA). Patients were stressed using adenosine for 6 minutes, and the stress ^82^Rb infusion was initiated 2.5 minutes after the start of the adenosine infusion (0.14 mg/kg/min). Low-dose CT for attenuation correction was performed before the rest study, and after the stress study if required. Coronary artery calcium score (CACS) images were acquired as per clinical routine from a noncontrast breath-hold CT. The CACS was calculated according to the Agatston score using a threshold of 130 Hounsfield units (HU).^16^

### Quantitative MBF and Flow Reserve

Both rest and stress dynamic images used for MBF quantification were reconstructed into 18 time frames (1 × 10 s, 8 × 5 s, 3 × 10 s, 2 × 20 s, and 4 × 60 s) on a 128 × 128 matrix, with 2 × zoom (voxel dimensions, 3.18 × 3.18 × 2.03 mm) using 3D OSEM reconstruction (2 iterations, 21 subsets) with point spread function modeling and time of flight.^17^ MBF quantification was performed using syngo software (Siemens Healthcare), which is based on a single-compartment model for ^82^Rb tracer kinetics.^18^ MFR was defined as MBF during maximal hyperemia divided by MBF during rest. The MBF at rest was corrected for baseline work by dividing MBF with the rate pressure product (RPP), which is the systolic blood pressure times the heart rate, multiplied by 10,000.^19^ MFR was divided into low (≤1.5), borderline (>1.5–2.0), and normal (>2.0).^12^

### Semiquantitative Analysis

The perfusion defects were computed automatically with Corridor4DM (INVIA, Ann Arbor, MI) as summed stress score (SSS) according to the AHA 17 myocardial segment model.^20^

### Left Ventricular Ejection Fraction

Corridor4DM was used for analysis of electrocardiographically gated data sets and for calculation of left ventricular ejection fraction (LVEF).

### Plasma Markers and Cardiovascular Risk Score

CD4 cell counts and HIV RNA levels were determined routinely on blood and plasma when collected.

Serum lipids were analyzed on a MODULAR ISE 1800 (Roche, Basel, Switzerland).

Framingham risk score (FRS) was calculated as the 10-year risk of coronary heart disease (CHD) according to published definitions.^21^

Statistics data are shown as mean ± standard error of the mean (SEM). Continuous variables were compared using unpaired *t* test after log_10_-transformation of variables necessary to obtain a normal distribution. Adjusted *t* test was performed in a multiple linear regression model. Correlations were analyzed using Spearman ρ on untransformed data. Categorical variables were compared by χ^2^ test. Test for interaction was performed with a general linear model. With a total of 81 patients in the 2 groups, we obtained a power of 0.8 to detect a difference of 0.5 in MFR (α = 0.05) using previously published standard deviation from cardiac perfusion data using ^82^Rb.^22^

All statistics were performed using SPSS 22 (IBM SPSS statistics for windows, version 22.0; Armonk, NY: IBM Corp). All data included in this article are available from the authors.

## RESULTS

The characteristics of the 2 groups are shown in Table 1. The groups displayed very similar cardiovascular risk factors a part from higher levels of both total cholesterol and high-density lipoprotein in the HIV-infected group.

**TABLE 1:**
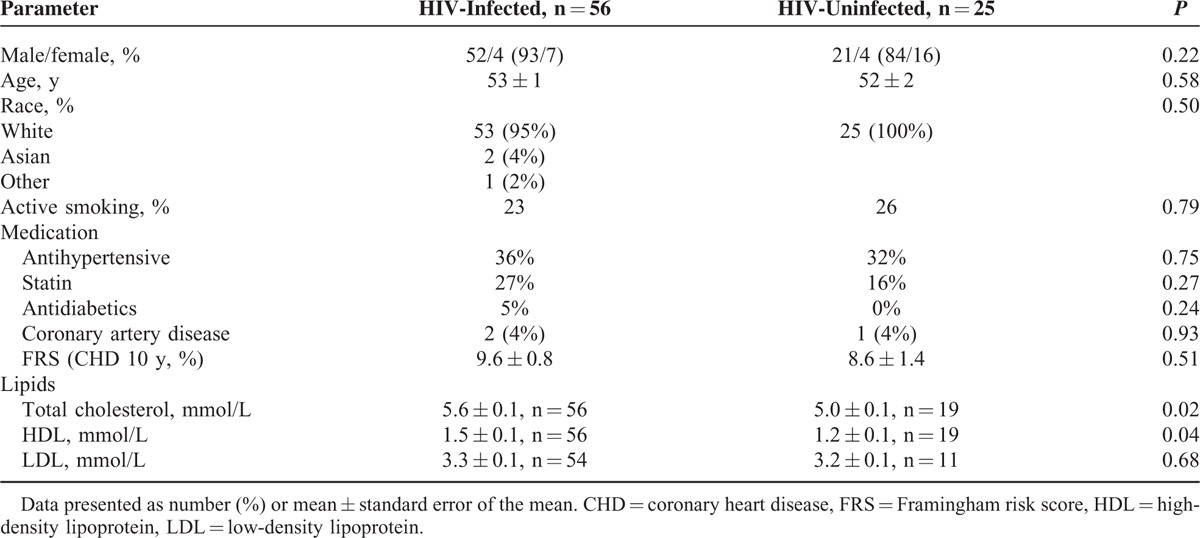
Baseline Characteristics

### HIV Parameters

All HIV-infected patients received ART with >90% of patients having CD4 cell counts >400 (10^6^/L), and all had viral loads <40 copies/mL. A total of 34/56 (61%) of the HIV-infected individuals received a protease-sparing regimen, whereas 34% received a regimen including at least 1 PI, and 48% received ABC (Table 2).

**TABLE 2:**
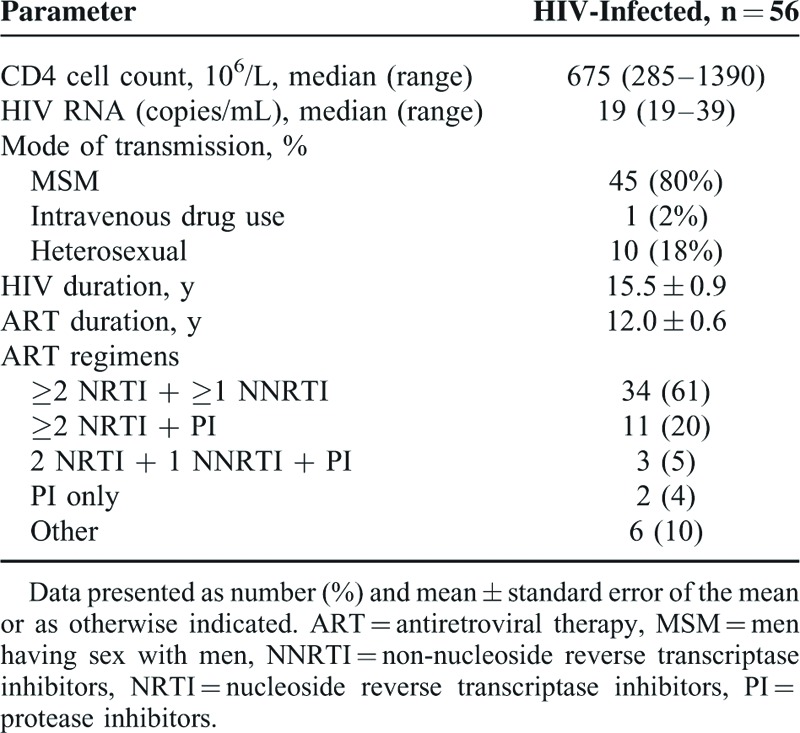
HIV-Related Characteristics

### Global MBF and MFR

**FIGURE 1:**
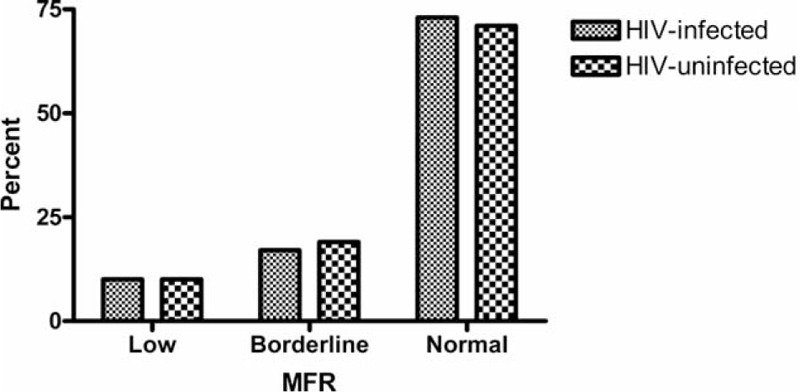
Percentages of HIV-infected patients and HIV-uninfected controls having low (<1.5), borderline (>1.5–2.0), and normal (>2.0) myocardial flow reserve.

Tables 3 and 4 summarize findings from the ^82^Rb PET MPI. MFR was comparable among HIV-infected and HIV-uninfected both with (2.52 ± 0.11 vs 2.37 ± 0.16; *P* = 0.44) and without correction for cardiac work at baseline (2.97 ± 0.11 vs 3.13 ± 0.17; *P* = 0.43). When MFR was divided into tertiles, HIV-infected and HIV-uninfected controls displayed very similar patterns (Fig. 1).

**TABLE 3:**
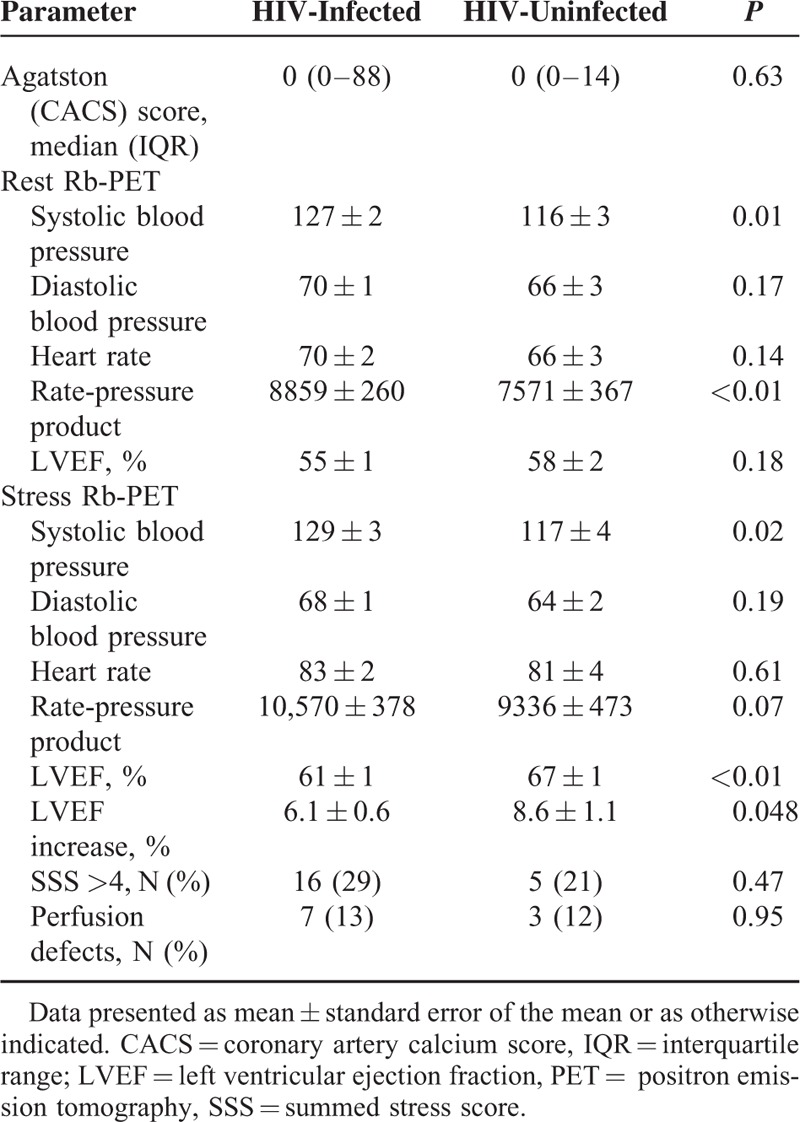
^82^Rb PET Data

**TABLE 4:**
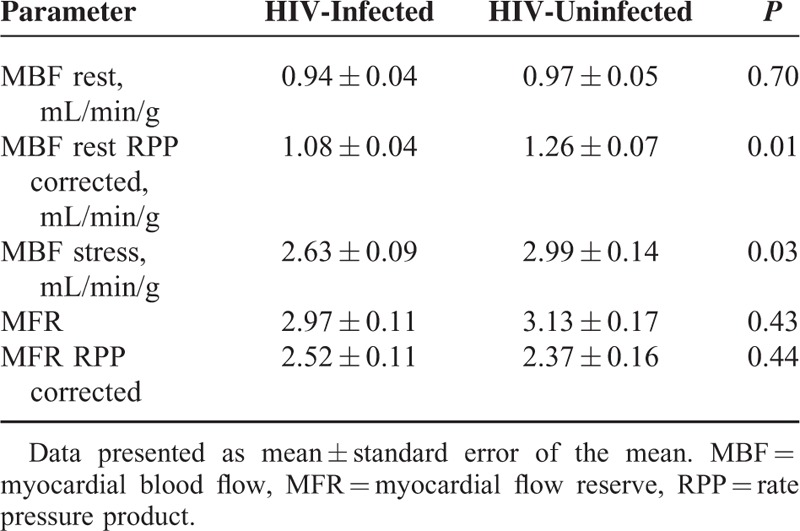
Quantitative Myocardial Perfusion Data

The resting MBF was comparable in the 2 groups, but after correction for cardiac work (RPP) the controls had higher MBF at rest. During stress, the MBF rose to higher levels among the HIV-uninfected controls. In an analysis excluding patients on antihypertensive medication and/or statin therapy, we found comparable values of MFR between the 2 groups both RPP corrected (2.62 ± 0.16 vs 2.39 ± 0.20; *P* = 0.39) and without correction (3.12 ± 0.18 vs 3.30 ± 0.22; *P* = 0.55). Adjusting for the difference found in lipid values between the 2 groups revealed no significant impact on MFR both with (*P* = 0.83) and without RPP correction (*P* = 0.31).

### Perfusion Defects

Perfusion defects were found among 13% of HIV-infected and 12% of HIV-uninfected (*P* = 0.95). The 3 patients, who at time of the study had known CAD, proved to have perfusion defects on the MPI and had the highest SSS (all ≥20).

### LVEF

A positive increase in LVEF during stress was found in all but 3 patients who were all HIV-infected. An increase of >5% in EF during stress^23^ was found among 80% in the HIV-infected group and 84% among healthy controls (*P* = 0.69). The mean increase in LVEF was lower among HIV-infected than controls (6.1 ± 0.6 vs 8.6 ± 1.1; *P* = 0.048). A positive correlation was found between the mean increase in LVEF and the MFR for all subjects (ρ = 0.27; *P* = 0.03) with no interaction of HIV status.

### HIV Parameters and MBF and MFR

**FIGURE 2:**
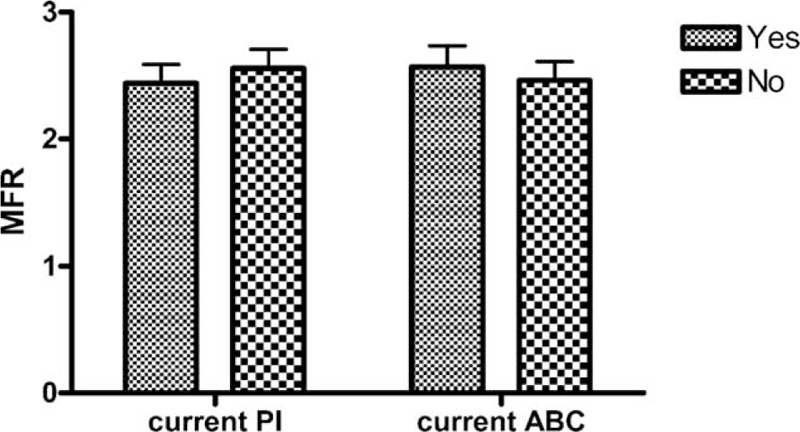
MFR for HIV-infected patients on current PI versus not on current PI, and HIV-infected patients on current ABC versus not on current ABC. ABC = abacavir, MFR = myocardial flow reserve, PI = protease inhibitors.

We found no correlation between MFR and CD4 cell counts (ρ = −0.19; *P* = 0.17), CD4 cell count at nadir (ρ = −0.20; *P* = 0.16), duration of HIV (ρ = 0.07; *P* = 0.64), or duration of ART (ρ = 0.08; *P* = 0.64). Current ART containing either PI or ABC did not influence MFR (Fig. 2).

## DISCUSSION

In this first study of MFR among HIV-infected patients using ^82^Rb PET, we found that levels of MFR were similar between HIV-infected patients with full viral suppression and HIV-uninfected controls indicating that well-controlled HIV infection does not compromise the function of the myocardial microcirculation significantly. Among HIV-uninfected patients suspected for CAD, the MFR has proven to be a highly predictive marker of future cardiovascular events.^12–14^ A normal MFR has a high negative predictive value for excluding high-risk CAD on angiography^24^ and an impaired MFR may even be better in selecting patients for revascularization than conventional angiography.^25^ In other groups of patients carrying a high risk of CVD, the MFR as assessed by PET predicts cardiovascular events in the absence of apparent epicardial stenosis.^26,27^ Among diabetic patients without overt CAD, an impaired MFR led to event rates of cardiac death comparable to those of patients with known CAD^27^ and in patients with chronic kidney disease MFR predicted cardiac death independently of traditional markers of clinical risk.^26^ In patients with lupus eythematosus or rheumatoid arthritis, who may share inflammatory risk factors with HIV-infected patients,^28^ a study of MFR found significantly lower values of MFR compared with healthy controls and that this reduction correlated with disease duration.^29^ In light of these data decreased values of MFR could have been expected among patients with HIV, as studies suggest that even on stable ART these patients may depict low-grade inflammation.^8^ On the contrary, we recently failed to find any signs of arterial inflammation as assessed by [18F]-2-fluoro-deoxy-D-glucose PET/CT of the arterial wall in a comparable cohort of HIV-infected patients.^30^ Together, our results may be indicative of a “normal” risk of CVD among HIV-infected patients in the Western world, where the focus on CVD risk reduction has increased in this population with resulting declining rates of MI.^31^

Few previous studies have quantified the absolute myocardial perfusion in HIV-infected patients. Our group was the first to do so by studying 13 HIV-infected patients with normal lipid values, 12 HIV-infected with dyslipidemia, and 14 healthy controls all nonsmokers assessing the impact of dyslipidemia on MBF and MFR using ^13^N-ammonia (^13^NH_3_) as PET tracer and no differences were found between the groups.^32^ In the present study, we consolidated that values of total cholesterol and HDL did not influence the MFR. Also, we have previously studied the impact of initiation of ART in 12 ART-naive HIV-infected patients by ^13^NH_3_ PET/CT and found that after 5 weeks of ART the stress MBF decreased by 31% and the MFR decreased by 20%^33^ indicating that ART may have a direct impact on coronary circulatory function. However, in this present study we found no difference between the groups suggesting that these changes are transient.

Previous studies have demonstrated impaired flow-mediated dilatation (FMD), another measure of endothelial function, in the brachial artery in HIV-infected patients. The impairment seems to be associated with both traditional risk factors^34^ and viral load.^35^ However, studies of ART and FMD have come to different conclusions.^4,36^ Interestingly, a recent, large study of FMD and carotid-intima media thickness of ART-naive patients found that ultrasonographic measures of CVD risk were more strongly associated with traditional risk factors than CD4 cell count, viral replication, and inflammatory markers.^34^ However, impairment of FMD of the brachial artery does not necessarily reflect deteriorations in the vasomotor function of the microcirculation in the myocardium.^37^ Other modalities for the assessment of arterial disease such as carotid-intima media thickness and pulse-wave velocity have indicated that HIV infection may affect both structural and functional properties of the arteries.^38^

Our group of HIV-infected patients had significantly lower values of stress MBF, which may indicate some influence on the myocardial microcirculation, despite the normal MFR. Still, no optimal threshold to define normal stress MBF for ^82^Rb has been reported so far.^39^ In a recent European study, the median value was 2.35 mL/g/min with a standard deviation of 0.88 mL/g/min using the same algorithm for perfusion analysis as in our study,^40^ indicating that the levels found in both groups of our study were well above “normal.” Still, it remains relevant to speculate whether the differences found in stress MBF could be caused by the ART and/or an impact on the vasodilator capacities during pharmacological stress using adenosine. Almost all of the HIV-infected patients received nucleoside reverse transcriptase inhibitors as part of their ART and several of them are purine analogues and could theoretically antagonize the function of adenosine by mechanisms similar to caffeine and other purine-based substances.^41^ Also, the use of recreational drug such as cocaine is reported to be higher among HIV-infected patients compared with the general population,^42^ and as cocaine has been shown to cause a decrease in myocardial perfusion,^43^ this could potentially influence myocardial vasomotor function in the HIV-infected group. The clinical relevance of impaired ability to respond to pharmacologically induced stress remains debated,^44^ and recent, large clinical studies assessing the predictive value of stress MBF and MFR have reached different conclusions on which parameter is most valuable using ^82^Rb as the PET tracer.^12,40,45^

Indeed, our group of HIV-infected patients had lower mean stress LVEF and lower rise in LVEF during stress than the healthy control group, but again, these values were within normal range^46^ except for the 1 HIV-infected patient with known cardiomyopathy.

### Limitations

The assessment of global MFR may overlook small changes within the segments of the myocardium, and further, patients with normal microcirculation could have subclinical atherosclerosis of the coronary arteries as a sign of early atherosclerosis as seen in large cross-sectional studies.^3^ This study is a single-center study reporting findings from one modality to assess coronary microvascular function, and we cannot exclude that small changes have gone overlooked. Accordingly, analyses of other biomarkers such as natriuretic peptides could have complemented our findings. This study was conducted in a cohort of mostly white HIV-infected patients with free access to ART and all patients were optimally treated, which may impair the ability to extrapolate to other HIV-infected populations.

## CONCLUSION

In this first study of MFR in HIV-infected patients on stable ART using ^82^Rb PET MPI, we found that patients had MFR comparable to healthy controls, which indicates that HIV infection or the ART does not impair the coronary microvascular function. In contrast, we did find lower values of stress MBF, but the significance of this finding remains unknown.
